# *LMNA* p.R482W mutation related to FPLD2 alters SREBP1-A type lamin interactions in human fibroblasts and adipose stem cells

**DOI:** 10.1186/1750-1172-10-S2-O13

**Published:** 2015-11-11

**Authors:** Brigitte Buendia

**Affiliations:** 1 Unité de Biologie Fonctionnelle et Adaptative (BFA), Université Paris Diderot-Paris 7, CNRS, UMR 8251, Paris, France

## 

SREBP1 (Sterol regulatory element binding protein 1), transcription factor that regulates hundreds of genes involved in lipid metabolism and adipocyte differentiation, is a direct partner of A-type lamins. We show that i) *in vitro*, the tail regions of prelamin A, lamin A and lamin C bind a polypeptide of SREBP1 and ii) within cells, interactions between wild-type A-type lamins and SREBP1 occur mainly at the nuclear periphery but also within the nucleoplasm. While A-type lamin R482W mutation is responsible for Dunnigan type familial partial lipodystrophy (FPLD2), we show that both overexpression of *LMNA* p.R482W in primary human preadipocytes and endogenous expression of A-type lamins p.R482W in FPLD2 patient fibroblasts, reduce A-type lamins-SREBP1 *in situ* interactions and upregulates a large number of SREBP1 target genes[1]. As this *LMNA* mutant was previously shown to inhibit adipogenic differentiation, we propose that deregulation of SREBP1 by mutated A-type lamins constitutes one underlying mechanism of the physiopathology of FPLD2.
